# Beyond intracranial pressure: Cerebrospinal fluid compartmentalization in spaceflight‐associated neuro‐ocular syndrome

**DOI:** 10.1113/EP093609

**Published:** 2026-02-15

**Authors:** Susan P. Mollan, Damian M. Bailey

**Affiliations:** ^1^ Department of Ophthalmology Queen's University Kingston Ontario Canada; ^2^ Institute of Metabolism and Systems Research, College of Medical and Dental Sciences University of Birmingham Birmingham UK; ^3^ Neurovascular Research Laboratory, Faculty of Life Sciences and Education University of South Wales Pontypridd UK; ^4^ Bexorg, Inc. New Haven Connecticut USA

1

There is no environment more physiologically extreme or challenging to humans than outer space (Bailey & van Ombergen, 2025). Astronauts are exposed to the full force of the space ‘exposome’, reflecting the cumulative burden of environmental stressors encountered during spaceflight, including cosmic radiation, isolation and confinement, distance from Earth, hostile and closed habitats, and altered gravity fields (Bailey et al., 2025). In combination, these interacting hazards place unprecedented strain on human physiological function and homeostasis. According to the National Aeronautics and Space Administration (NASA) Human Research Program (NASA, 2023), they are associated with >30 identified risks to human health, several of which are classified as high priority or ‘red‐rated’ owing to both their likelihood and potential severity, spanning acute in‐mission threats to consequences emerging later in life (Romero & Francisco, 2020). Understanding, integrating and mitigating these risks remains a central challenge for space agencies and commercial partners alike (Bailey, 2025).

Among these hazards, spaceflight‐associated neuro‐ocular syndrome (SANS) has emerged as one of the most concerning and least mechanistically resolved red risks. Since its initial description (Mader et al., 2011), SANS has been recognized as a complex clinical syndrome characterized by decrements in near visual acuity, hyperopic refractive shifts, optic disc oedema, posterior globe flattening, choroidal and retinal folds, cotton wool spots and alterations in retinal nerve fibre layer thickness, often presenting asymmetrically. These changes typically develop after several weeks of microgravity exposure and, in some astronauts, persist long after return to Earth, raising serious concern for exploration‐class missions, in which visual impairment could directly compromise crew safety and mission success.

A central paradox underpins SANS. Within the orbit, clinical features resemble those of elevated intracranial pressure (ICP), including optic disc oedema, globe flattening and chorio‐retinal folds. Yet intracranial findings tell a different story, with lumbar puncture opening pressures frequently normal or only borderline elevated, accompanied instead by cephalad fluid redistribution, upward brain shift and ventricular expansion (Ng & Mollan, 2025). Reconciling these discordant ocular and cerebral signatures has proved challenging.

Because these observations diverge fundamentally from terrestrial disorders, such as idiopathic intracranial hypertension, the underlying pathophysiology remains unresolved. Does SANS arise from elevated ICP, from CSF compartmentalization or from an interaction between the two? This uncertainty is not merely conceptual. Countermeasures targeting ICP reduction, venous congestion or fluid redistribution have largely evolved in parallel rather than within an integrated physiological framework. Consequently, monitoring strategies and therapeutic development remain fragmented, limiting progress in mitigating one of the most mission‐critical risks of long‐duration spaceflight, namely the threat of sustained visual impairment during a crewed mission to Mars.

In this issue of *Experimental Physiology*, the paper by Tang et al. (2026) provides an important step change in unifying the neuro‐ophthalmological features of SANS. They posed a deceptively simple but physiologically intuitive hypothesis that CSF redistribution, rather than uniform elevation of ICP, underpins the coupled structural changes observed across the brain–eye axis in microgravity. To test this, they implemented a prospective cross‐sectional study design involving both Roscosmos cosmonauts and European Space Agency (ESA) astronauts, alongside matched terrestrial control participants at each study site.

Using high‐resolution brain magnetic resonance imaging acquired within the ESA‐endorsed prospective ‘BRAIN‐DTI’ programme, Tang et al. (2025) deployed automated morphometric analysis to quantify brain–eye relationships, focusing specifically on correlations between third ventricle volume and detailed ocular parameters. A key methodological strength was the use of the validated MReye‐Seg pipeline, enabling three‐dimensional volumetric characterization of ocular tissues (Tang et al., 2025). This represents a major conceptual and technical advance over traditional two‐dimensional optic nerve sheath diameter measurements, which are known to be insensitive to subtle regional and compartment‐specific changes and have yielded inconsistent findings.

At baseline, the authors established that the volume of the optic nerve sheath complex, representing a direct extension of the intracranial CSF space, was correlated with third ventricular volume. Additional orbital structures, including optic nerve length, retro‐orbital width and globe dimensions, also demonstrated significant baseline relationships to third ventricle volume. These findings provide the first quantitative evidence in humans that normal brain–eye anatomy is intrinsically coupled through CSF‐mediated interactions even under terrestrial gravity, thereby establishing an essential physiological reference framework against which spaceflight‐induced perturbations can be interpreted.

Following spaceflight, the expected increases in optic nerve sheath volume and optic nerve length were observed. Crucially, however, third ventricle expansion was correlated selectively with changes in globe volume and globe length, while dissociating from other orbital changes. The magnitude of third ventricle expansion was specifically associated with posterior globe deformation and reduced globe volume, which are structural hallmarks directly implicated in hyperopic refractive shifts. This selective coupling provides compelling evidence that microgravity induces differential biomechanical effects across orbital compartments, rather than transmitting pressure uniformly throughout the cranial–orbital system.

A trend towards a reduction in optic nerve sheath volume during early post‐flight recovery was observed, whereas third ventricle expansion persisted when measured within 14 days after return to Earth. This divergence in recovery trajectories is particularly informative, suggesting that distinct CSF compartments might normalize at different rates and further supporting the concept of compartment‐specific CSF regulation rather than a single homogeneous pressure system.

The structural changes in the eye were noted to be asymmetric. Although this might appear surprising, optic disc oedema secondary to raised ICP is frequently asymmetric in terrestrial disease (Mollan, 2025). What is striking in the context of SANS, however, is that such asymmetry emerges in the absence of sustained global intracranial hypertension. This again argues against a purely pressure‐driven model and instead implicates regional differences in CSF flow, tissue compliance and venous outflow within the orbit.

Intracranial pressure itself is often conceptualized as a static variable, yet in reality it is highly dynamic, being influenced by posture, respiration, arterial pulsatility, venous drainage, brain compliance, autoregulation and, uniquely in spaceflight, the near‐complete removal of gravitational hydrostatic gradients. Recent advances in neurophysiology suggest that ICP alone might be an incomplete descriptor of CSF physiology. Increasing attention has therefore shifted towards CSF pulsatility and flow dynamics as potential drivers of tissue deformation and symptom development.

Indeed, abnormal CSF pulsatility is increasingly recognized as a pathological determinant in several neurosurgical conditions, including Chiari malformation and normal‐pressure hydrocephalus, where clinical manifestations arise despite normal or only modestly elevated ICP (Tsermoulas et al., 2026). In this context, the findings by Tang et al. (2025) provide a powerful physiological parallel: microgravity might alter CSF flow pathways and compliance relationships, generating regionally amplified mechanical stresses at vulnerable interfaces, such as the posterior globe and lamina cribrosa, without necessitating sustained elevation of ICP.

Overall, this study provides quantitative, structure‐specific evidence supporting CSF compartmentalization and a limited intracranial expansion (‘cranial ceiling’) model as central mechanisms in SANS. By demonstrating preserved ventricle–globe coupling alongside dissociation from optic nerve sheath and retro‐orbital changes, Tang et al. (2025) move the field beyond the long‐standing dichotomy of ‘raised ICP versus venous congestion’. Instead, their work supports a more physiologically integrative framework, in which microgravity induces a cranial fluid redistribution that differentially loads anatomical compartments according to local compliance, geometry and drainage capacity.

This conceptual advance is not merely academic. It provides a crucial physiological foundation for next‐generation countermeasures, suggesting that effective mitigation of SANS will require strategies that address CSF dynamics, compartmental flow and tissue biomechanics, rather than global pressure reduction alone. As humanity prepares for prolonged lunar habitation and crewed missions to Mars, such mechanistic clarity represents an essential step towards safeguarding one of the most mission‐limiting vulnerabilities of the human nervous system, namely vision.

## AUTHOR CONTRIBUTIONS

Susan P. Mollan and Damian M. Bailey contributed to the conception of the Viewpoint. Each author participated in designing, writing and revising the Viewpoint, approved the final version and agrees to be accountable for all aspects of the work in ensuring that questions related to the accuracy or integrity of any part of the work are appropriately investigated and resolved. Both persons designated as authors qualify for authorship, and all those who qualify for authorship are listed.

## CONFLICT OF INTEREST

S.P.M. reports consultancy fees (NovoNordisk); advisory board fees (Ocular therapeutix, Dompé); speaker fees (Teva, AbbVie); Travel (AbbVie, Essilorluxottica, European Alliance of Associations for Rheumatology); and receipt of equipment (Heidelberg Engineering). D.M.B. is Editor‐in‐Chief of *Experimental Physiology*, outgoing Chair of the Life Sciences Working Group, a member of the Human Spaceflight and Exploration Science Advisory Committee to the European Space Agency, a member of the Space Exploration Advisory Committee to the UK and Swedish National Space Agencies and a member of the National Cardiovascular Network for Wales and South‐East Wales Vascular Network.
